# Commentary: Fc Gamma Receptors are Expressed in the Developing Rat Brain and Activate Downstream Signaling Molecules upon Cross-Linking with Immune Complex

**DOI:** 10.29245/2572.942X/2019/1.1243

**Published:** 2019-02-18

**Authors:** Marianna Stamou, Pamela J Lein

**Affiliations:** 1 Department of Molecular Biosciences, School of Veterinary Medicine, 1089 Veterinary Medicine Drive, University of California, Davis, CA 95616; 2 ETH Zurich, Department of Health Sciences and Technology, Institute of Molecular Systems Biology, 8093 Zürich, Switzerland

During the past 20 years, a number of studies have reported IgG antibodies against viral or self-antigens in the developing human brain. Although the mechanisms by which they gain access to the developing brain are not yet clear, a subset of these antibodies has been linked to increased risk for neurodevelopmental disorders^1–11^. At first glance, such effects are expected to be mediated by IgG binding to its cognate Fcγ receptors (FcγR) on resident immune cells (such as microglia) in the brain and subsequent activation of local innate immune responses. However, considering that antibodies linked to neurodevelopmental disorders have subsequently been shown to recognize intracellular antigens expressed in neurons and astrocytes^7^, it is tempting to hypothesize that these autoantibodies could derail normal neurodevelopment by binding to their target antigens expressed on non-immune cells in the brain. Neuronal uptake of IgG antibodies against intracellular neuronal antigens has previously been shown and, in some cases, is thought to be mediated via clathrin-dependent endocytosis of IgG bound to FcγR in these neurons^12, 13^. In combination with recent studies showing functional expression of FcγRI in adult rat dorsal root ganglion neurons^14–16^, these findings prompted us to conduct the first comprehensive investigation of FcγR expression and signaling on neurons and astrocytes in the developing rat brain. In our study, we documented sex-independent *in vivo* expression.

In our study we documented sex-independent *in vivo* expression of FcgRIa, *FcgRIIa, FcgRIIb, FcgRIIIa*, and *Fcgrt* mRNA in the neonatal (postnatal days 1 and 7) rat cortex, hippocampus, and cerebellum at levels comparable to those observed in the spleen. Based on these findings, we investigated the functional expression of these FcγR *in vitro* using primary co-cultures of neurons and astrocytes from neonatal rat pups. In both hippocampal and cortical cultures, we confirmed protein expression of FcγRIa, FcγRIIb, and FcγRIIIa but not FcRn in both cell types and showed that, similar to immune cells, expression of these receptors at the mRNA level is regulated by IFNγ. Antibody-antigen IgG immune complexes (IgG-IC) triggered increased [Ca^2+^]_i_, a small but statistically significant increase in Erk phosphorylation, and FcγR endocytosis in these cells, consistent with canonical FcγR signaling in immune cells^17–21^. Calcium responses were concentration-dependent but showed high variability, consistent with [Ca^2+^]_I_ transient variability in immune cells depending on cell type, activation state and ratio of excitatory to inhibitory FcγR on the cell surface^22, 23.^ The variability in [Ca^2+^]_i_ responses can be further explained by our findings of subsets of neurons and astrocytes expressing either the excitatory FcγRI, or the inhibitory FcγRIIb or both. Although we were not able to detect intracellular IgG in neurons or astrocytes that endocytosed FcγR following crosslinking with IgG-IC, inhibition of FcγR endocytosis by pre-incubation with anti-FcγRIa or anti-FcγRIIb antibodies further supports that FcγR signaling in immature neurons and astrocytes is triggered in the presence ofimmune complexes. Collectively, our findings of IFNγ-regulated functional FcγR expression by developing neurons and astrocytes underscore the need for further investigation into the role of Fcγ receptors during normal and aberrant neurodevelopment.

The strength of our experimental approach is the functional characterization of neuronal/astrocytic FcγR using IgG-IC to crosslink FcγR on the cell surface and activate downstream signaling. Although IgG glycosylation is an important regulator of IgG-mediated immune signaling, monosaccharide IgG antibodies can also mediate effector functions in immune cells via FcγR, but *in vitro* these functions can be predicted only when immune complexes are used^24^. In addition, with the exception of FcγRI, which can be activated by monomeric IgG, all other IgG subclasses are activated in the presence of immune complexes but not by monomeric IgG (with the exception of FcRn, which functions to protect monomeric IgG from degradation)^18, 25^. To promote activation of all FcγR isoforms in our study, and considering that we had no information regarding the glycosylation state of the IgG used to generate the immune complexes, we pre-incubated mouse IgG with rat anti-mouse IgG to generate IgG-IC across all our functional studies.

Despite this strength, there are potential gaps in our findings. Although the downstream effects we observed in our study (increased [Ca^2+^]_i_, Erk phosphorylation and FcγR endocytosis) were triggered in the presence of IgG-IC, the IgG-IC concentration used to observe endocytosis was above physiologically relevant levels documented in rat cerebrospinal fluid (100 μg/ml; although in humans such levels would be considered low)^26–28^, while similarly high concentrations of monomeric IgG also triggered [Ca^2+^]_i_ and Erk phosphorylation but not FcγR endocytosis. At the same time, the exact composition of the IgG-IC (ratio between rat IgG subclasses, IgG glycosylation state, and size of the immune complex) was not characterized and could have varied between individual preparations, thus affecting FcγR crosslinking, activation and downstream signaling^24, 26, 29^. Another consideration is that the Fc portion of IgG can also be recognized by non-classical Fc-binding receptors on the cell surface. Such receptors have been described in immune cells (FcR-like 5 (FcRL5), Dectin-2, DC-Sign, MMR and MBL2)^30^, but the expression of such molecules in neurons/astrocytes in the developing brain has not yet been investigated. In summary, the specificity of FcγR involvement in the IgG-IC-induced cellular signaling should be confirmed by knocking down FcγR expression in these cells; this would also be useful for further assessing the specificity of the commercially available antibodies used to probe FcγR protein expression in these cells. Studies of IgG-IC-mediated activation of additional signaling molecules downstream of FcγR should also be conducted to identify alignment with or potential deviations from canonical immune cell FcγR signaling, which would further confirm a functional role of FcγR in developing neurons/astrocytes. As an example, activation of neuronal FcγRI in rat DRG neurons (which express FcγRIa but not FcγRIIb) results in increased [Ca^2+^]_i_, by triggering the non-selective cation channel TRPC3 through the Syk-PLC-IP_3_ pathway^15, 16^. Although we probed for Syk phosphorylation in rat hippocampal and cortical neurons, we were not able to demonstrate Syk expression in these cells (unpublished data). The possibility that manual application of compounds to cultured neurons may trigger mechanosensitive receptor-mediated [Ca^2+^]_i_, increase^31^, thus partially contributing to the observed effects, cannot be excluded. In addition, considering that hippocampal and cortical neurons express activating and inhibitory FcγR on their surface, isoform-specific FcγR knockdown and careful probing of downstream effectors would be useful in dissecting the relative contribution of each isoform on the downstream pathways activated^32–34^.

But what if the true role of neuronal/astrocytic FcγR lies in bidirectional signaling with resident microglia and other immune cells in the brain? Expression of classical immune molecules such as MHC Class I proteins and complement receptors has been demonstrated to regulate microglia-mediated synaptic pruning during neurodevelopment^35–37^. Generating conditional neuronal FcγR knockout models would be instrumental in assessing the existence of analogous interactions between neuronal FcγR and microglia, and in understanding the role of neuronal FcγR *in vivo*.

Going back to the original question of whether autoantibodies against intracellular brain antigens could derail normal neurodevelopment by engaging their intracellular targets via FcγR-mediated uptake, there are additional questions that need to be addressed. Are these autoantibodies taken up by developing neurons in a non-stochastic manner? Are these autoantibodies already complexed with their target antigens when internalized? If not, is there another mechanism for their uptake into neurons which does not involve FcγR engagement? Are these autoantibodies function-blocking? Alternatively, considering that non-specific monomeric IgG may be neuroprotective^26^, could this be the case also for maternal autoantibodies with specific brain antigen targets? Or are these autoantibodies simply diagnostic? Would neuronal subtypes with prevalent FcγRI expression be more susceptible to uptake and effects of these antibodies? How would endocytosed IgG escape lysosomal degradation in neurons? Further studies addressing these questions are needed in order to assess a potential role for FcγR in developing neurons and astrocytes in the reported association between maternal antibodies targeting developing brain antigens and increased risk for neurodevelopmental disorders.

In summary, hippocampal and cortical neurons express Fcγ receptors and IgG immune complexes seem to crosslink these receptors, resulting in activation of downstream signaling. Although the functional readouts of IgG-IC priming of these cells are part of the immune cell signaling cascade normally activated by FcγR crosslinking, further studies are needed to further elucidate the neurodevelopmental consequences of FcγR expression in developing neurons and astrocytes and probe their potential activation by maternal autoantibodies against brain antigens.
